# Lattice Boltzmann Model of 3D Multiphase Flow in Artery Bifurcation Aneurysm Problem

**DOI:** 10.1155/2016/6143126

**Published:** 2016-04-28

**Authors:** Aizat Abas, N. Hafizah Mokhtar, M. H. H. Ishak, M. Z. Abdullah, Ang Ho Tian

**Affiliations:** ^1^School of Mechanical Engineering, Universiti Sains Malaysia, Engineering Campus, 14300 Nibong Tebal, Penang, Malaysia; ^2^School of Aerospace Engineering, Universiti Sains Malaysia, Engineering Campus, 14300 Nibong Tebal, Penang, Malaysia

## Abstract

This paper simulates and predicts the laminar flow inside the 3D aneurysm geometry, since the hemodynamic situation in the blood vessels is difficult to determine and visualize using standard imaging techniques, for example, magnetic resonance imaging (MRI). Three different types of Lattice Boltzmann (LB) models are computed, namely, single relaxation time (SRT), multiple relaxation time (MRT), and regularized BGK models. The results obtained using these different versions of the LB-based code will then be validated with ANSYS FLUENT, a commercially available finite volume- (FV-) based CFD solver. The simulated flow profiles that include velocity, pressure, and wall shear stress (WSS) are then compared between the two solvers. The predicted outcomes show that all the LB models are comparable and in good agreement with the FVM solver for complex blood flow simulation. The findings also show minor differences in their WSS profiles. The performance of the parallel implementation for each solver is also included and discussed in this paper. In terms of parallelization, it was shown that LBM-based code performed better in terms of the computation time required.

## 1. Introduction

Aneurysm is a condition in which a blood vessel wall is pathologically dilated. It normally happens at the vicinity of the circle of Willis, the main cerebral arteries, and has also been found to develop in the iliac and intracranial arteries [1]. Aneurysm could lead to artery rupture resulting in stroke or even death. The arterial wall may rupture when the wall itself is not strong enough to withstand the stresses exerted on it during the blood flow. As the treatment methods for aneurysm carry a high degree of risk [2], understanding the vessel's internal fluid flow is important. It can be utilized to improve aneurysm diagnosis and treatment methods. Hashimoto et al. [3] pointed out that the aneurysm growth can be associated with the intra-aneurysmal hemodynamics. Hence, good understanding and knowledge of hemodynamic parameters are crucial. Various computational fluid dynamics (CFD) studies [4–9] have been carried out to investigate the relationship between hemodynamic factors and the risk of an artery wall's rupture. These factors were the impingement force, the WSS, the pressure, and the blood velocity.

For the past two decades, the simplicity and the effectiveness of the Lattice Boltzmann method (LBM) in solving flow related problem has created awareness in the CFD community. LBM-based computations have been applied to simulate different complex and irregular geometries presented in flow problems. Some of these experiments demonstrated that LBM performed better than the classical CFD tools in certain applications [10–12]. The LB simulation is based on the Boltzmann theory instead of the expensive and time-consuming Navier-Stokes equation. LBM did have its limitations, despite the increasing popularity in solving flow problems. For example, though LBM is capable of solving high-Mach number flow in aerodynamics, it still lacks a consistent thermohydrodynamic scheme. However, some efforts were concerted to sort out these limitations [13].

The past few years have seen many researchers resorting to LBM in an attempt biomedical problem. In the work by Liu, biviscosity constitutive relations and control dynamics based on a D2Q9 lattice are used to solve a stenosis blood flow problem [14]. Sun and Munn predicted the blood flow by developing an LBM model that considers the blood as a suspension of particles in the plasma [15]. In addition, Leitner et al. introduced new LBM boundary conditions for the cardiovascular domain using a D2Q9 lattice model [16]. The application of LBM to three-dimensional (3D) problems is rather limited and noticeable applications can be found in the work by [17], featuring a 3D simulation of intracranial aneurysm geometry and in the work by [18] on wall orientation and shear stress in the artery.

In general, the application of LBM is not restricted to the fluid flow simulation. It has been found to be an efficient solver in sound absorption applications [19]. Another interesting use is in the simulation of floating rigid bodies on a free surface [20]. The LBM and the FVM solver can be combined together to solve the rigid body problem. Following this development, various other simulations were conducted utilizing this coupling method [4, 21–23].

Vast amount of performance analysis and optimization techniques has been found conducted to utilize the computation efficiency of LBM software. Kopta et al. [24] and Tian et al. [25] try to evaluate the parallel performance of LBM in terms of its scalability and efficiency. In addition, a number of optimization steps can be constructed on the LB code to improve the simulation efficiency [26]. Normally, OpenMPI and OpenMP are the two principle programming models often used in high-performance computing (HPC). The parallelization usually includes a pure message-passing-interface (MPI) algorithm that can be used along with LBM solver. A study has been found that combines both of these models in an effort to reduce the computation resources [26], especially involving complex geometries. Parallel performance can be further improved by replacing the CPU with Graphics Processing Units (GPU) algorithms during numerical calculation. GPU was originally developed for computer games and will provide better computation power for scientific applications compared to the conventional CPU unit [27].

In the current work, the prediction and the investigation of the complex, unsteady blood flow inside aneurysm geometry is explored by using LBM-based software, Palabos. To optimize the results obtained, three different collision models, namely, the incompressible BGK model, the regularized BGK model, and the multiple relaxation time (MRT) model, are compared to investigate the efficacy of each method in modelling blood flow problem [28]. The accuracy of these different LB-based scheme in solving aneurysm blow flow simulations will be examined by comparing the hemodynamic parameters with the conventional finite volume method based solver. To the best of the authors' knowledge, there is no study conducted to compare the capability of both FVM and LBM models typically in the simulation blood flow problems in terms of the solution accuracy and parallel performance. The current study attempts to compare the distribution of mesh for both LBM and FVM models in the detection of high wall shear stress (WSS) region in the arterial wall. The parallel speedup with increasing number of cores is also compared between both LBM and FVM solvers in terms of its parallel efficacy.

## 2. Lattice Boltzmann Models

The results shown in this section are formulated using D3Q19 lattice model. The LBM equation can be summarized in

(1) $$\begin{array}{c} f\left(\;r+cdt,c+Fdt,t+dt\;\right)-f\left(\;r,c,t\;\right)=\Omega f\left(\;r,c,t\;\right), \end{array}$$

in which the right-hand side represents the streaming step. The left-hand term denotes the collision term which can be represented using the well-known Bhatnagar-Gross-Krook (BGK) model as given in

(2) $$\begin{array}{c} \Omega =\omega \left(\;f^{\text{eq}}-f\;\right)=\frac{1}{\tau }\left(\;f^{\text{eq}}-f\;\right). \end{array}$$

*ω* and *τ* denote the relaxation frequency and time. *f* ^eq^ represents the equilibrium function that relates to the lattice arrangement.

The equilibrium function, *f* ^eq^, can be described as

**Figure 1:**
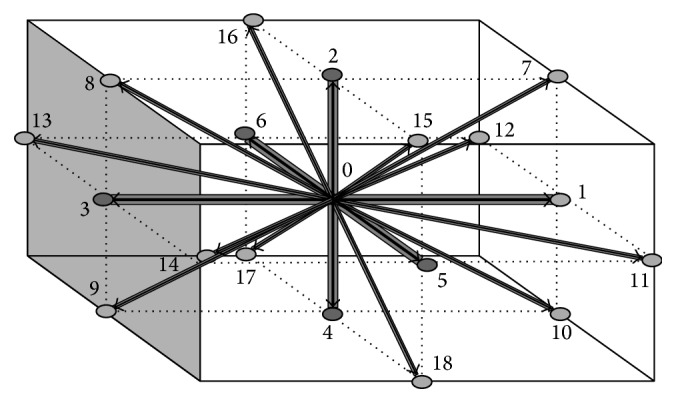
3D lattice arrangements for D3Q19.

(3) feqρ,u=ρw1+1cs2c·u+12cs4c·u2−12cs2u·u

in which *w* represents weighting function across different lattice links. For the case of D3Q19 lattice model as depicted in Figure 1, the weighting functions can be described in Table 1.

Microscopic velocities for a D3Q19 lattice model are given as

(4) e0=0,0,0,e1,2=±1,0,0,e3,4=0,±1,0,e5,6=0,0,±1,e7–10=±1,±1,0,e11–14=±1,0,±1,e15–18=±1,0,±1.

### 2.1. Conventional BGK Collision Model

The underlying theory of LBM is based on the discrete Boltzmann equation. Due to the complicated collision term that exists in the RHS of the Boltzmann equation, it is difficult and burdensome to solve. To ease the computational effort, Bhatnagar et al. [32] proposed a simplified version of the collision operator. The collision operator can be replaced as

(5) $$\begin{array}{c} \Omega =\omega \left(\;f^{\text{eq}}-f\;\right)=\frac{1}{\tau }\left(\;f^{\text{eq}}-f\;\right), \end{array}$$

where *ω* = 1/*τ*. The coefficient *ω* denotes the collision frequency and *τ* is the relaxation factor. *f* ^eq^ is the Maxwell-Boltzmann equilibrium distribution function. By substituting the approximate collision operator, the discrete Boltzmann equation can be shown as

(6) $$\begin{array}{c} f_{i}\left(\;\vec{x}+\vec{e_{i}},t+1\;\right)=f_{i}\left(\;\vec{x},t\;\right)+\Omega , \end{array}$$

where *i* is the index selected between the possible discrete velocity directions and *e*_*i*_ is the direction of the selected velocity.

The fluid density and macroscopic velocity can be found from the moment of the distribution function as shown below:

(7) ρ=∑ifi,u→=1ρ∑ifiei→.

Subsequently, the equilibrium distribution, *f* ^eq^, can be arranged according to the Maxwell-Boltzmann distribution form as

(8) $$\begin{array}{c} f_{i}^{\text{eq}}\left(\;\vec{x},t\;\right)=w_{i}\rho \left[\;1+3\left(\;\vec{e_{i}}\vec{u}\;\right)+\frac{9}{2}{\left(\;\vec{e_{i}}\vec{u}\;\right)}^{2}-\frac{3}{2}{\vec{u}}^{2}\;\right]. \end{array}$$

Equation (5) will replace the commonly used Navier-Stokes equation in CFD simulations. It is also possible to derive N-S equation from Boltzmann equation using the Chapman-Enskog model.

### 2.2. Incompressible BGK Model

In the fundamental form of BGK, the equilibrium term is multiplied by the fluid density as shown in (5). In incompressible model, the density term, *ρ*, will be formulated as *ρ* = *ρ*_0_ + *δρ* where *ρ*_0_ is a constant value and *δρ* is the infinitesimal changes in density. The equilibrium distribution function then becomes [16]

(9) $$\begin{array}{c} f_{i}^{\text{eq}}\left(\;\vec{x},t\;\right)=w_{i}\left[\;\rho +\rho_{0}\left\{\;3\left(\;\vec{e_{i}}\vec{u}\;\right)+\frac{9}{2}{\left(\;\vec{e_{i}}\vec{u}\;\right)}^{2}-\frac{3}{2}{\vec{u}}^{2}\;\right\}\;\right] \end{array}$$

in which all terms containing $\vec{u}$ are multiplied by the constant, *ρ*_0_. Since the velocity is defined to be proportional to the momentum, the incompressible model is computationally cheaper than the standard BGK as it avoids the division by *ρ* in (3). It also reduces the compressibility errors introduced by the quasi-incompressible nature of LBM.

### 2.3. Regularized BGK Model

The regularized model can be extended from the standard BGK dynamics with relatively simpler precollision steps based on the study by Latt and Chopard [33]. The use of this model can increase numerical stability with less consumption of the computational overhead. The equilibrium term *f* ^eq^ reads

(10) $$\begin{array}{c} f_{i}^{\text{eq}}=\rho t_{i}\left[\;1+\frac{v_{i\alpha }u_{\propto }}{c_{s}^{2}}+\frac{1}{2c_{s}^{4}}Q_{i\propto \beta }u_{\propto }u_{\beta }\;\right], \end{array}$$

where a repeated Greek index implies a summation over this index. The tensors *Q*_*i*∝*β*_ are defined to *Q*_*i*∝*β*_ = *v*_*iα*_ *u*_*iβ*_ − *c*_*s*_^2^ *δ*_∝*β*_, and *t*_*i*_'s as well as *c*_*s*_ are the coefficients specific to the lattice topology.

### 2.4. Multiple Relaxation Time Model (MRT)

The linear collision operator is recast into the space of velocity moments and their corresponding relaxation parameters are individually adjusted to improve numerical stability or to modify the physics of the model. In the MRT model, the local discrete Maxwellian function [34] can be approximated as

(11) f∝eq,Mρ,u→=w∝1+e∝→·ρu→RT+e∝→·ρu→22RT2−12u→·ρu→RT,

where *w*_∝_ is the weighting factor. In this representation of the local Maxwellian and the factor RT is related to the speed of sound, *c*_*s*_, of the model through RT = *c*_*s*_^2^, where *c*_*s*_ = 1/√3*c*.

### 2.5. Finite Volume Method

FVM simulation has served as a viable alternative to experimental methods and is more superior in terms of its ability to determine flow variables which are difficult to obtain in experiments. The basic concept of CFD problems mostly involved solving the Navier-Stokes (N-S) equation. FVM is one of the most commonly used approaches in CFD and recent progress in computational capability has led to the use of FVM in solving various critical medical problems. These medical cases typically for blood flow problems often require complex simulation that demands substantial memory usage and high calculation speed. Many researchers have utilized N-S equation in solving flow related problems of aneurysm cases with highly reliable simulation results produced using finite volume based numerical method [35–50]. In FVM, the N-S equations comprise continuity, mass, and energy conservation equations. The simplified version of the finite volume equation for fluid flow yields the governing equations as described in (12) and (13)

*Continuity Equation. *Consider the following:

(12) $$\begin{array}{c} \frac{\partial \rho }{\partial t}+\nabla \cdot \rho \vec{u}=0. \end{array}$$

*Momentum Equation. *Consider the following:

(13) $$\begin{array}{c} \frac{\partial }{\partial t}\rho \vec{u}+\nabla \cdot \left(\;\rho \vec{u}\vec{u}\;\right)=-\nabla P+\nabla \cdot \overset{̿}{\tau }+\rho \vec{g}. \end{array}$$

## 3. Parallelization: Message Passing Interface (MPI)

Parallelization is performed via the message-passing-interface (MPI) paradigm of the MPI library. It is a standardized portable MPI system that works on a wide variety of parallel computer platforms. MPI helps to subdivide large problem into smaller and more manageable ones that are equally distributed for each processor in the parallel system. Each processor will then solve the problem concurrently and the results are combined at the end of the parallelization. The main function of MPI is to speedup the execution of application by subdividing the job among all multiple processing elements in parallel device. Exploitation of all processors or cores in parallel machines is necessary to ensure significant speedup in calculation.

## 4. Multiphase Formulation

**Figure 2:**
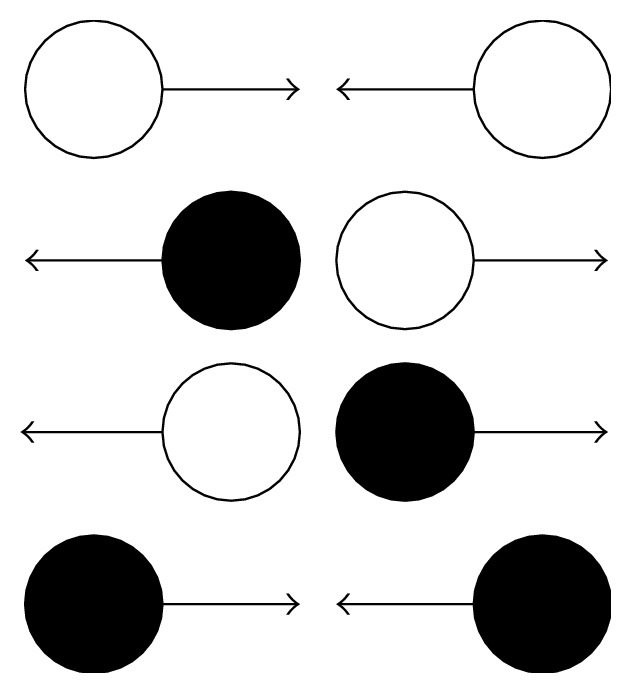
The meaning of pseudopotential force. Each lattice population experiences an attractive force from color-mates neighbours and a repulsive one from the opposite color.

Shan et al. introduced pseudopotential model for multicomponent [51] and multiphase flow [52, 53] as depicted in Figure 2. It was a popular model based on collision operator that has been improved by long-range interaction force that imitates the pairwise interaction potential between dissimilar phases or components and their findings have shown that impressive results are obtainable with fairly minimal effort. The Shan-Chen model is therefore highly popular, despite potential numerical deficiencies in some areas [52]. The Shan-Chen model represents the source term as follows:

(14) $$\begin{array}{c} V\left(\;x,x^{\prime }\;\right)=G\left(\;x,x^{\prime }\;\right)\psi \left(\;x\;\right)\psi \left(\;x^{\prime }\;\right), \end{array}$$

where *G* is a Green's function and *ψ* = *ψ*(*p*) is the “effective mass.” Given the potential, the interaction force is written as

(15) $$\begin{array}{c} F=-G\left(\;\left|\;e_{\propto }\;\right|\;\right)\psi \left(\;x\;\right)\underset{\alpha }{\sum }\psi \left(\;x+e_{\alpha }\;\right)e_{\alpha }. \end{array}$$

The strength of interaction is controlled by the amplitude parameter *G*. Note that positive *G* means attraction meanwhile negative *G* means a repulsion. As a result, phase separation is determined by positive entries in the diagonal interaction matrix *G* and negative (repulsion) on the off-diagonal.

For most practical purposes, this interaction is described in its “corral” form as

(16) $$\begin{array}{c} G\left(\;\left|\;e_{\propto }\;\right|\;\right)=\left\{\begin{array}{cc} G, & \text{if }\left|e_{\alpha }\right|=c,\\ 0, & \text{if }\left|e_{\alpha }\right|>c, \end{array}\right. \end{array}$$

where *c* is the lattice spacing. The velocity *u* ^(eq)^ is changed to *u* + *τF*/*ρ* when the equilibrium distribution function is computed. The averaged momentum before and after collision is *ρu* + *F*/2. The nonideal gas equation of state (EOS) was specified by *ψ*(*ρ*) = *ρ*_*o*_[1 − exp⁡(−*ρ*/*ρ*_*o*_)]. Analytical calculation can be computed to find the value of *G* and *ρ*, where the value of *G* is determined by the temperature. For the same fluid, the coexistence of two densities presents at a range of *ρ* where *dp*/*dρ* is negative for the case that is below the critical values. The advantage of using this model in phase segregation is generated automatically. The trade-off however is that the collision operator does not conserve momentum locally and instead momentum is conserved globally if there is no momentum exchanged occurring at the boundaries.

## 5. Methodology

### 5.1. Simulation Setup

**Figure 3:**
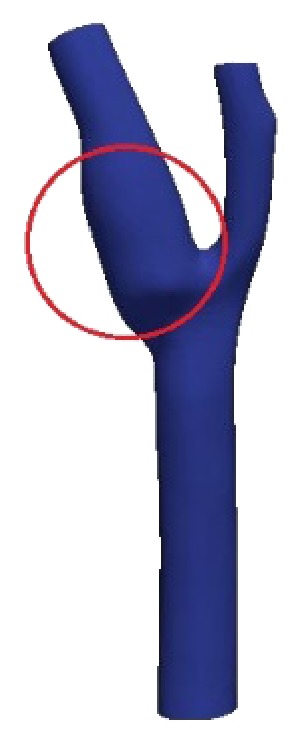
Artery bifurcation aneurysms geometry.

An artery bifurcation aneurysm geometry located at the branch point of the common, internal, and external carotid arteries is created using the CAD software as shown in Figure 3. The carotid arteries function as blood vessel that carries blood from the neck to the brain region. El-Sabrout and Cooley found out that the occurrence of aneurysm in the extracranial carotid artery is found to be rare [54]. Their research also revealed that in most cases aneurysm in the external portion is seldom reported and limited research has been conducted to study the treatment procedure since it involves diverse etiologies and present diagnostic and therapeutic challenges. However, this aneurysm affected carotid artery, if left untreated, may cause neurologic symptom from embolization or serious complications that include rupture and thrombosis [55, 56]. Various treatment methods are recommended for patients with unruptured intracranial aneurysm as early precaution to future aneurysm rupture with varying risk depending on age, smoking, and size of the aneurysm [29]. Johnston et al. found that post detection of intracranial aneurysm includes rupture or subarachnoid hemorrhage, associated with 32%–67% fatality and 10%–20% long term effect at the specified brain area [57]. Their study also shows that, for ruptured aneurysm, early detection or study on the intracranial region, within 24 to 72 hours, can help to reduce the chance of subsequent subarachnoid hemorrhage. In a separate study, it was reported that 50% of rupture also occurs in the aneurysm affected iliac artery region with 50% to 70% mortality rate [58]. The common iliac artery originates from the abdominal aorta that is the main blood vessel in the abdominal area. Given the fatality rate associated with aneurysm cases, it is imperative that the aneurysm be detected at the onset of its development and intervention steps begin [30].

The carotid artery aneurysm is chosen in this paper for future development of stent design studies in the treatment of aneurysm that may cause serious complications to the wall artery. The bifurcation region of the intracranial artery comprising common, internal, and external carotid arteries is chosen with the range of diameters given as 0.43–0.77 cm, 0.394–0.53 cm, and 0.36–0.56 cm, respectively [59–61].  Table 2 summarizes the differences between the intracranial/cerebral and iliac arteries with respect to the vessel caliber and wall thickness. Incidentally, these different wall dimensions will incur different stress levels on the artery wall that can be detected using numerical simulation. The treatment procedure can then be proposed depending on the distribution of wall stresses that is highly dependent on the branching angle and flow type.

Norma et al. study the development factor of aneurysm that includes role of bifurcation, age, gender, and environmental factor. It was shown that, for study conducted on elderly age older than 65 years old, the prevalence of aneurysm is approximately 5% to 6% in men while 1% to 2% for women. The study has also shown the importance of branching angle study with acute angle branch, commonly attributed with smokers and aging factors, could give rise to turbulence and increased in wall shear stress [62]. By referring to the research by Rossitti and Löfgren, their mathematical formulation has shown that the blood flow rate influences the wall shear stress proportional to the third power of the vessel radius. These changes in blood flow rate will continuously change the vessel radius resulting in induction of high stress region at certain parts of the artery wall [63]. In Figure 3, the carotid artery branch model consisting of one inlet (common artery) and two outlets (external and internal arteries) with different diameters is created using CAD model [64]. The inlet diameter is approximately 0.63 cm, while the first and the second outlet diameters are about 0.45 cm and 0.3 cm, respectively.

**Figure 4:**
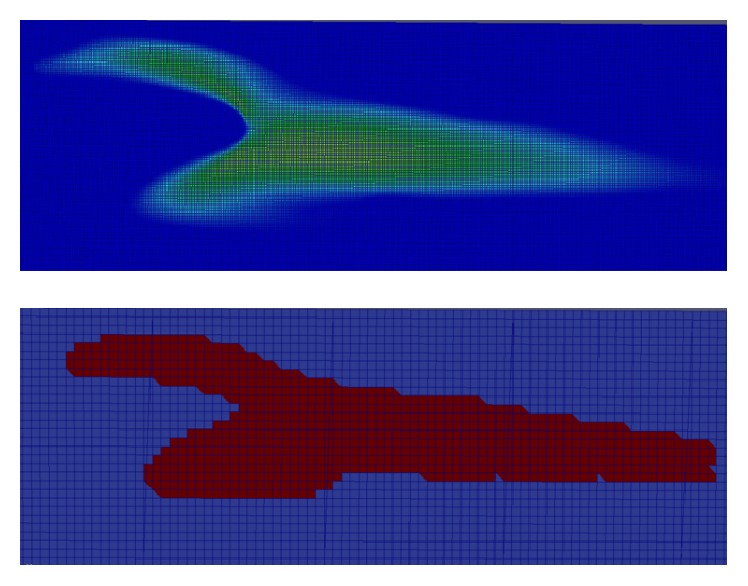
Regular mesh in LBM model at particular plane.

Mesh generation is one of the most critical parts in fluid simulation. The quality of the mesh will determine the accuracy and the stability of the numerical calculation. The triangular mesh setup was generated using both of the LB and FV methods. The mesh creation process of Palabos is written in the source code and the mesh is automatically generated when the Palabos code is compiled and run. The constructed mesh using the LBM approach is all regular and no additional complexity is required to create such mesh.  Figure 4 shows an example of blood flow model that has been discretized into regular mesh. The upper model has a higher resolution as compared to the lower model. The interfaces and the fluid properties in each cell are calculated and solved using the Lattice Boltzmann equation.

**Figure 5:**
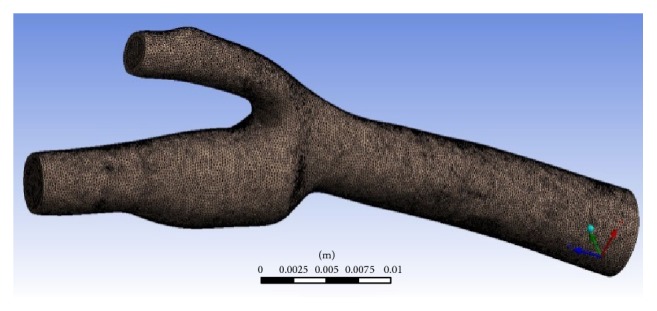
Mesh created by external software in ANSYS FLUENT.

In comparison, FLUENT needs external meshing software to produce the volume mesh. This volume mesh generation process is structured carefully to ensure reliable simulation results. Moreover, the simulation results are strongly dependent on the final mesh as well.  Figure 5 depicts an unstructured tetrahedral mesh generated using ANSYS FLUENT.

To optimize the LBM simulation, three different types of solvers were chosen, to compare their accuracy against that of the FVM solver. They are the incompressible BGK model, the regularized BGK model, and the multiple relaxation time (MRT) model. In FLUENT, the pressure-based type solver is selected. The pressure-based approach was developed for low-speed incompressible flows and is compatible with the current blood flow problem. In each simulation, the blood is modelled as a Newtonian fluid to ensure the physical properties remain consistent throughout the experiment.  Table 3 shows the blood flow parameters used in this simulation.

A constant parabolic velocity profile is imposed on the inlet with zero pressure set at both outlets. The wall boundaries are also imposed using nonslip boundary condition. The average velocity is set at 0.1 m/s in this research according to blood flow velocity in artery used to investigate aneurysm cases based on the experiment and simulation from research outcome by Gabe et al. [65]. Since the inlet velocity profile in FLUENT is constant, a user-defined file (UDF) of the parabolic profile is created to standardize both simulations. The UDF parabolic profile is created according to the Power Law Laminar formula = 2*u*_avg_[1 − (*r*/*R*)^2^], where *R* is the inlet radius and *u*_avg_ denotes the average velocity over the cross section. In a recent study [53], the parabolic formulation produced highly similar results compared to the CFD simulation for cases involving the effect of the inlet velocity profile using real-patience-specific inlet velocity profile. By combining all the parameters to the inlet diameter, the flow's Reynolds number was estimated at around 170.

The physical properties of a fluid must be converted into the LB discrete system during LBM implementation. There are constraints that must be adhered to in determining the choice of the lattice variables. The first principle is that the discrete system must be equivalent to the physical system; for example, Reynolds number of incompressible flow must give the same value. Another constraint is that the parameters should be fine-tuned in order to reach and maintain the required accuracy and stability during simulation run. For example, the grid should be sufficiently resolved, or the discrete time step should be small enough to ensure smooth simulation computation process. In this simulation, the discrete space interval of *δx* can be obtained from the aneurysm geometry and the discrete time step, *δt*, is chosen as another fixed variable. The discrete space interval can be defined as the reference length divided by the number of cells, *N*, used to discretize the length. The resolution, *N*, is set at 350 with the reference length set at 0.05589 m corresponding to *δx* = 0.000156563. Since the physical time step is set as 0.01 s, *δt* is discretized as 0.00005. The same physical time step is also applied in the FLUENT solver. With two known variables, *δx* and *δt*, other variables such as discrete average velocity, *u*_LB_, discrete kinematic viscosity, *v*_LB_, and relaxation time, *τ*, can now be easily transformed between the physical and the LB system as shown in

(17) uLB=uphysical×∂t∂x,vLB=vphysical×∂t∂x2,τ=3vLB+0.5.

Variables *u*_LB_ and *v*_LB_ were calculated as 0.0319361 and 0.00755492, respectively, with *τ* = 0.52266. This relaxation that is approaching 0.5 yields a stable simulation using double precision arithmetic during the computations. The final step is conducted to validate the first principle through Reynolds number in lattice units. The Reynolds numbers in LB unit can be calculated via

(18) $$\begin{array}{c} Re=\frac{u_{\text{LB}}N_{\text{inlet}}}{v_{\text{LB}}}, \end{array}$$

where *N*_inlet_ is the lattice nodes of the inlet with value given by *N*_inlet_ ≈ 19.36477 lattices nodes. The calculated Reynolds number is then a value of around 170.

### 5.2. Parallel Computation

A comparative study is conducted to compare the efficacy of parallel implementation scheme in the LBM and the FVM scheme in a multiprocessor computer, Quad Cores Intel® Xeon® CPU W3520@2.67 GHz with a 12-GB installed memory (12-GB ram). The system type is 64-bit Microsoft Windows XP operating system. MPI was used as the message passing library to provide a straightforward interface to write a software program that can utilize multiple cores of the computer. A program written using Palabos can be automatically parallelized through the use of the MPI library. Major improvement in the speed up could be seen when all of the cores in a parallel system are being exploited highlighting the importance of parallel architecture. In contrast, in FLUENT, a built-in MPI system is included and ready for use. To ensure similar setup between LBM and FVM, the number of lattice elements of meshing was set to almost similar value for the two geometries. The mesh information of the fluid zone domain in FLUENT comprised 1866320 elements and 455001 nodes, whereas in LBM it comprised 1728430 elements and 487359 nodes. For comparison, only the incompressible BGK model was selected as the LBM collision method to be compared to the FLUENT simulation.

## 6. Results and Discussion

### 6.1. Comparisons between Methods

**Figure 6:**
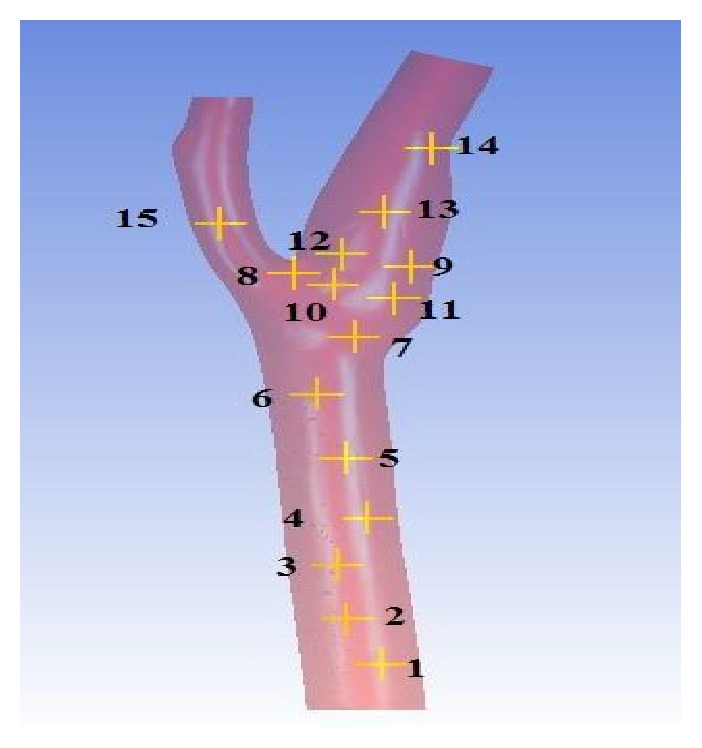
Coordinates of 15 points of interest.

**Figure 7:**
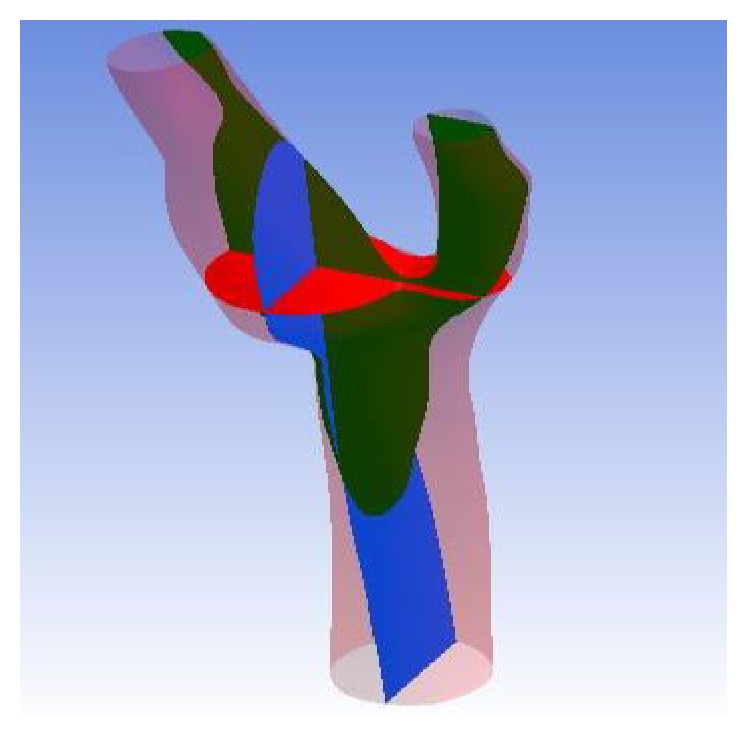
Orientation of 3 planes (*XY* plane is red, *YZ* plane is blue, and *ZX* plane is green).

**Figure 8:**
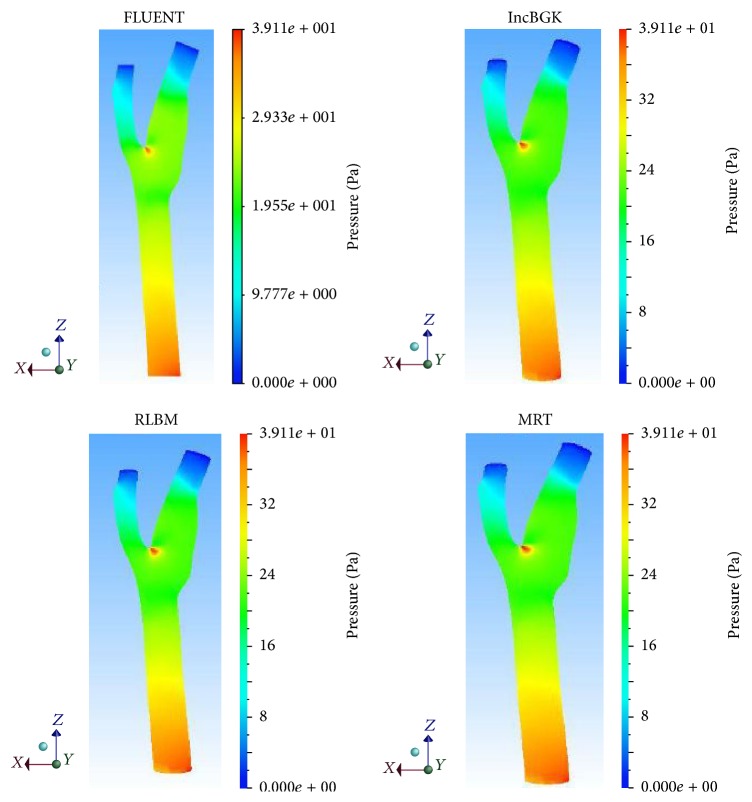
Pressure contours in the different simulations at the wall artery of aneurysm geometry.

The solutions were observed and analyzed after running the aneurysm case on both solvers. The wall artery of aneurysm geometry itself along with additional 15 points of interest and three planes has been used to explore the blood flow reaction. The dissimilarity of these two solvers is also investigated.  Figure 6 shows the fifteen coordinate points distributed throughout the geometry and Figure 7 depicts the orientation of the planes. The position of points and orientation of planes is chosen in this way so that every part of the flow can be well understood and studied. The monitoring process started with the pressure profile when the steady state of the flow is reached.  Figure 8 compares the pressure contours of four different methods in wall artery. The pressure contours of incompressible BGK model (IncBGK), regularized BGK model (RLBM), and multiple relaxation time model (MRT) are almost identical compared to the FVM model. All four pressure contours show that pressures are high at the vicinity of the inlet region and the pressure gradually decreases until it reaches zero value at the two outlets.

**Figure 9:**
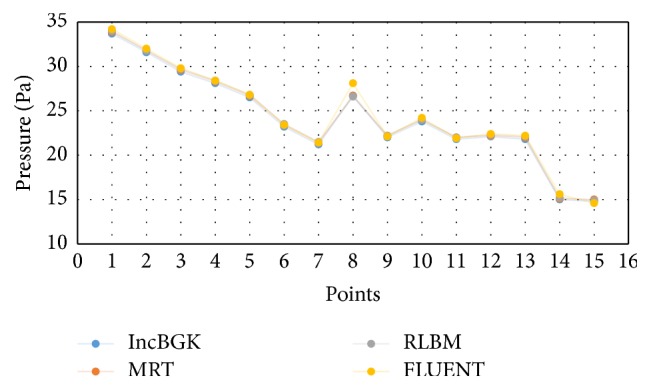
Comparison of the pressure of points of interest between LBM and FVM models.

**Figure 10:**
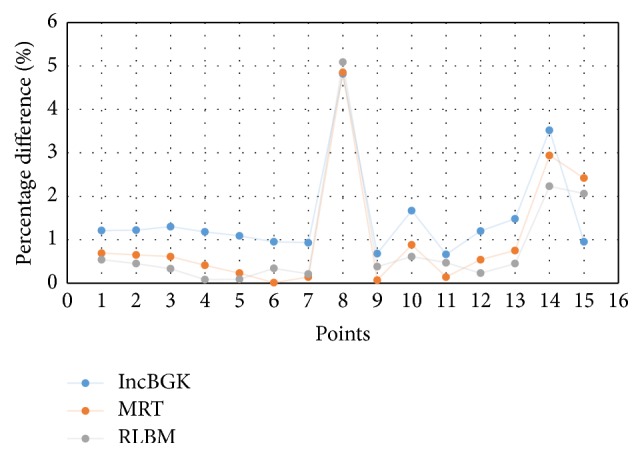
Percentage difference (pressure) at each point of interest for all LBM models relative to FVM.

The blood flow problem is related to the Poiseuille flow and there must exist some pressure difference as the blood flows through the artery. Another likeliness of the four models is the high pressure spot found in the bifurcation region. This is probably due to the collision of the bloodstream and wall as the stream separates into two branches resulting in high pressure spot. The pressure of each appointed point is compared among the solvers and graphed in Figure 9.  Figure 10 displays the percentage difference of pressure and these differences are computed from

(19) $$\begin{array}{c} \text{Percentage Diff}=\left|\;\frac{P_{\text{LBM}}-P_{\text{FVM}}}{P_{\text{FVM}}}\;\right|\times 100\%. \end{array}$$

All LBM models give high degree of accuracy relative to the FVM model. In terms of the pressure profile, the maximum percentage difference is of just merely 5% for comparison at point 8. Furthermore, Figure 7 shows that all lines are overlapping with each other. Thus, it can be concluded that LBM model is able to produce comparable results with FVM model in terms of the pressure profile.

**Figure 11:**
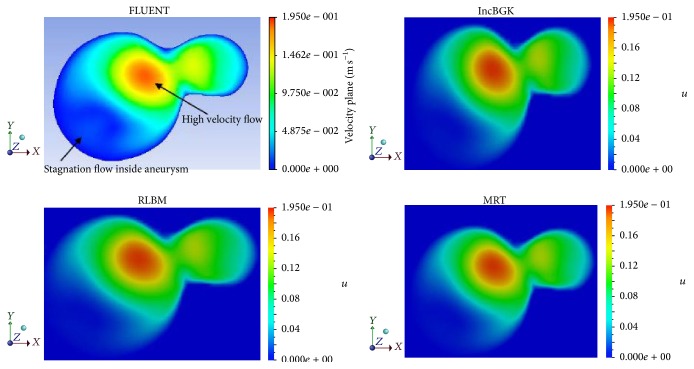
Velocity magnitude contours at *XY* plane for different simulations.

**Figure 12:**
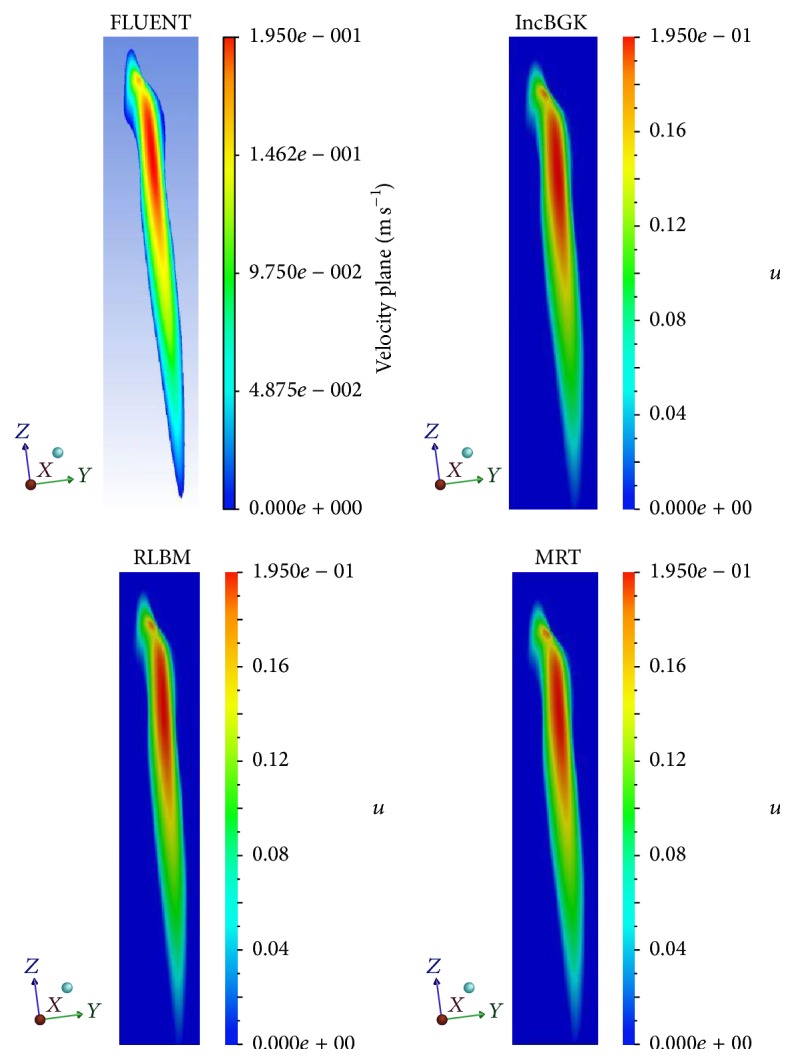
Velocity magnitude contours at *YZ* plane for different simulations.

**Figure 13:**
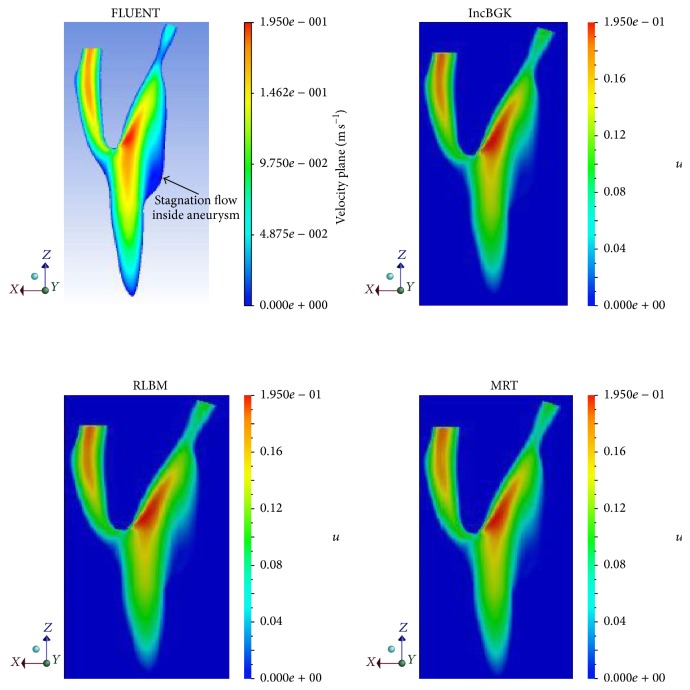
Velocity magnitude contours at *ZX* plane for different simulations.

For the velocity profile, the velocity magnitude contours for different simulations were placed side by side and compared according to their respective cross section. Figures 11, 12, and 13 show the velocity contours in *XY*, *YZ*, and *ZX* plane.

From Figures 11 to 13, it was revealed that the characteristics of the flow field are similar for different simulation methods at the given cross section. All LBM models have almost identical velocity contours between each other although some minor deviation can be found for the solutions obtained using FVM. As depicted in Figure 11, the stagnation flow is observed inside the aneurysm for all simulation since the velocity is almost zero. There are high velocities of flow stream detected near the bifurcation point for every simulation. The high velocity flow is believed to have hit the wall causing high pressure spot as previously demonstrated in Figure 8. The same characteristic of flow can be predicted from *ZX* plane in which the high velocity can be observed near the bifurcation region while zero velocity region is visible inside the aneurysm. Similarly, for the pressure profile, the velocity magnitude at each interest point location will be compared to determine the accuracy between these two methods. The plot of velocity magnitude and percentage differences is compiled in Figures 12 and 13.

**Figure 14:**
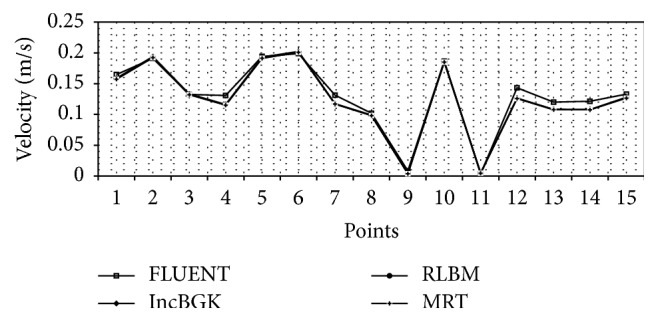
Comparison of the velocity of 15 points of interest between LBM and FVM models.

**Figure 15:**
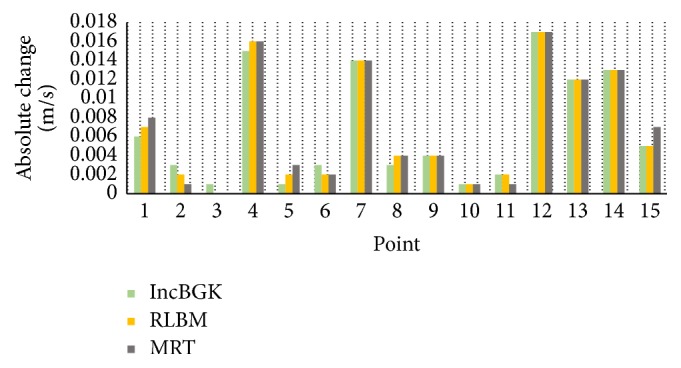
Absolute change (velocity) at each point of interest for all LBM models.

Referring to Figure 14, the velocity magnitude at each location for FLUENT, incompressible BGK LBM model, multiple relaxing time LBM model, and regularized BGK LBM models shows small variation and almost overlaps with each other.  Figure 15 shows the “absolute change” of velocity for all LBM models relative to the FLUENT FVM model. The absolute change in velocities for all LBM models relative to the FVM model is computed from

(20) $$\begin{array}{c} \text{Absolute Change}=\left|\;V_{\text{FVM}}-V_{\text{LBM}}\;\right|. \end{array}$$

Note that at point 3, RLBM and MRT have similar values of velocities relative to the FVM models. In comparison, for all points, the velocity values of all LBM models show minor differences relative to the FVM model. This demonstrates the capability of all LBM models compared to the conventional established FVM model.

**Figure 16:**
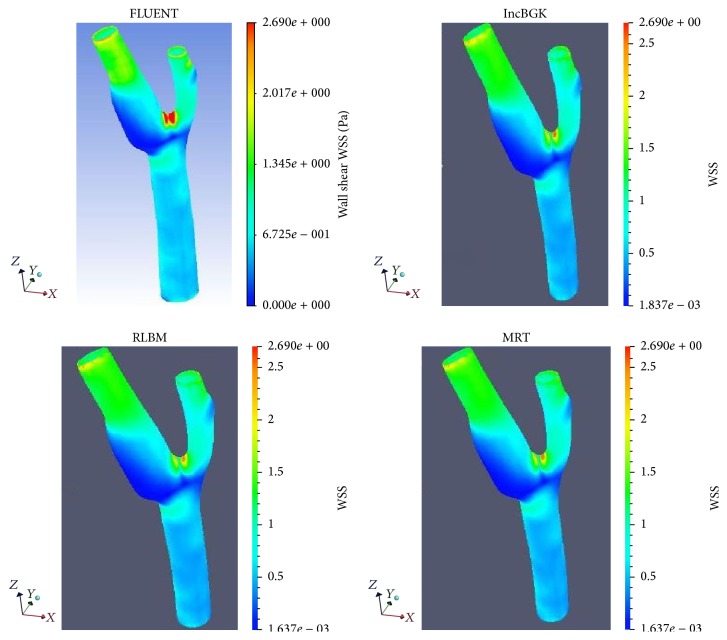
Wall shear stress in the different simulations at the wall artery of aneurysm geometry.

Wall shear stress is a flow-induced stress that can be described as the frictional force of viscous blood. It has always been thought of as the most important parameter for clinical diagnosis of artery disease. It is also one of the most reliable indicators for discriminating the rupture point of the aneurysm geometry. Two theories can be used to explain the mechanism of aneurysms rupture, which are high-flow and low-flow theories. Consistent with these theories, some CFD studies came out with different findings regarding the rupture status of aneurysm. In a study by Bai-Nan et al. [62] and Cebral et al. [66], it was reported that the ruptured aneurysms are associated with higher wall shear stress but recent studies pointed out that the rupture point of aneurysm is induced by remarkably low shear stress [8, 67]. In our simulation, we found that both highest and lowest wall shear stress exist in the geometry. The highest wall shear stress (WSS) occurs near to the bifurcation region whereas the lowest wall shear stress is observed in the aneurysm wall. These two areas may possess the highest risk of rupture since there exist two regions of highly concentrated stresses. These high stress regions may pose problem if they exceed the allowable stress level causing the wall to deform. Subsequently, it will lead to aneurysm deformation or in worst case scenario can lead to the rupture at the artery wall. From Figure 14, both LBM and FVM solutions can precisely indicate the area of highest and lowest WSS. The FVM model tends to have higher WSS at the vicinity of the bifurcation point. As demonstrated previously in Figures 8 and 16, the highest WSS area also corresponds to relatively high pressure and velocity. This is the region where the bloodstream persistently hit the walls thereby causing this high WSS values.

**Figure 17:**
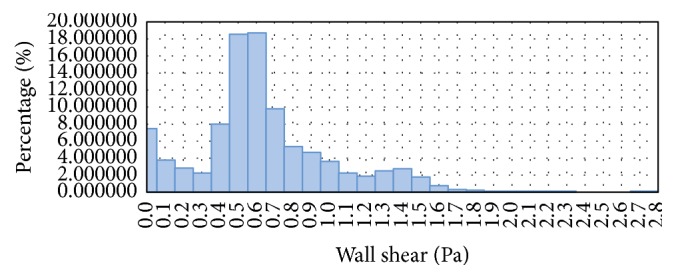
Histogram of percentage of mesh number at different WSS values for FVM.

**Figure 18:**
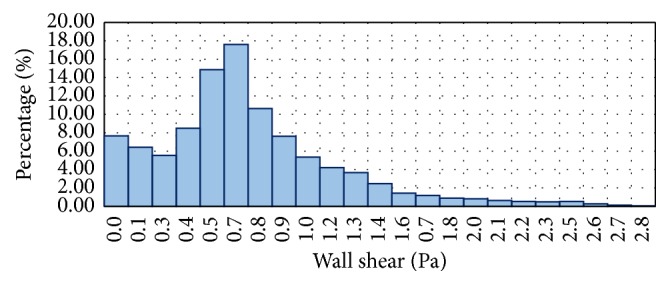
Histogram of percentage of mesh number at different WSS values for LBM.

In order to compare the capability of LBM and FVM solver, the histogram of WSS profile at wall artery of geometry is studied and analyzed. Only FLUENT and incompressible LBM model were compared since the remaining LBM models gave comparatively similar results. The WSS histograms for both FVM and LBM are shown in Figures 17 and 18, respectively.

**Figure 19:**
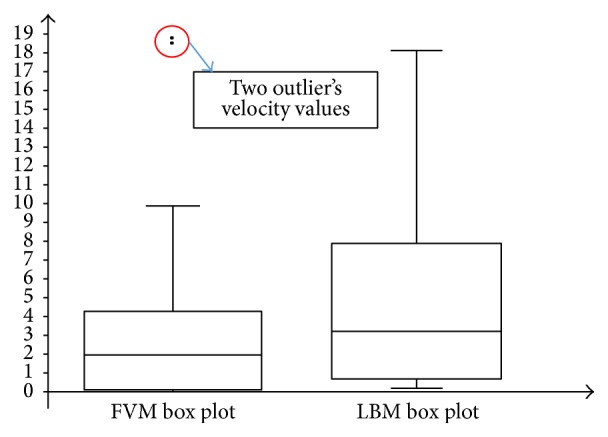
Box plot of LBM and FVM models for percentage of mesh number between WSS range of 0–2.8 Pa.

In comparison, the WSS profile for LBM shows relatively better distribution of mesh compared to FVM model. This is due to the distribution of mesh in LBM as shown in Figure 18 which is decreasing more uniformly from one peak value to the other indicating highly continuous distribution of numerical approximation across all of the mesh. For the FVM model as depicted in Figure 17, sudden peak particularly at the points with WSS value of 1.4–1.5 Pa and low concentration of mesh distribution for WSS value in the range of 1.8–2.8 Pa demonstrates the lack of continuity from on mesh to the other. To complement this statement, two box plots were constructed as depicted in Figure 19 based on the percentage of mesh number between WSS values of 0–2.8 Pa to compare the variability and uniformity of the current mesh distribution in LBM and FVM models. From Figure 19, two outlier values of velocities can be seen in the FVM model with the box plot having minimal variation of data between the first and third quartile indicating less uniform percentage of mesh distribution. In contrast, it was shown that no outliers were observed in the LBM with considerably better variation of velocity data ranging from 0.5 to 7.5 Pa between the first and the third quartiles. This spread of data shows good uniformity for LBM method mesh distribution moving from one WSS value to the other.

Since the treatment method is dependent on the distribution of WSS, highly uniform mesh typically at the high WSS region as depicted in Figure 18 for the LBM model can accurately identify the critical WSS region to determine the best possible treatment options. Early studies have pointed out that morphological changes in the vessel wall may result in growth of aneurysm over time [68–71]. Aneurysm rupture can occur if the arterial wall is unable to withstand the increase in hemodynamic forces [70]. It is believed that the low-WSS segments can weaken the arterial wall structure and high WSS can lead to aneurysm wall destructive modelling [72]. Therefore, treatment strategies of aneurysm could be conducted to reduce risk of rupture. For small, unruptured, and asymptomatic aneurysm, observation of the wall artery using image scanning can be conducted every year with no surgical treatment advised. However, for high risk aneurysm that may lead to rupture, it could be treated by surgical clipping or endovascular treatment such as stent placement, coil embolization, and flow diversion [73, 74].

### 6.2. Multiphase Blood Flow Simulation

**Figure 20:**
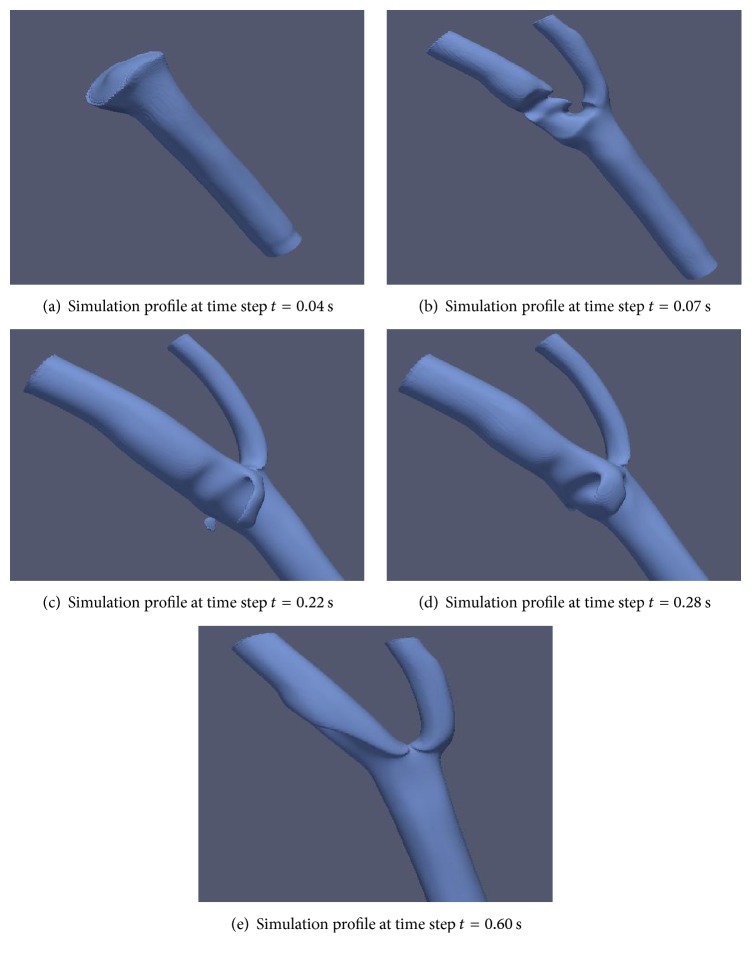
3D multiphase blood flow for velocity more than 0.05 m/s at different time steps.

In this section, the impact of high velocity 3D flow model towards the artery wall is observed. The 3D flows with velocity higher than 0.05 m/s will be visualized to examine its impact on the artery wall. The multiphase simulation profile of blood flow with velocity more than 0.05 m/s at various time steps is shown in Figure 20.

The blood will stream and get separated into two main branches. Noticeable nonvisible region can be observed on the left side of the branch. This region with velocity less than 0.05 m/s will exhibit the highest wall shear stress (WSS). WSS is a dynamic frictional force induced by viscous fluid moving along a surface of solid material. It is highly important that this region of high WSS is diagnosed earlier before it bursts as a result of significant buildup of WSS.

**Figure 21:**
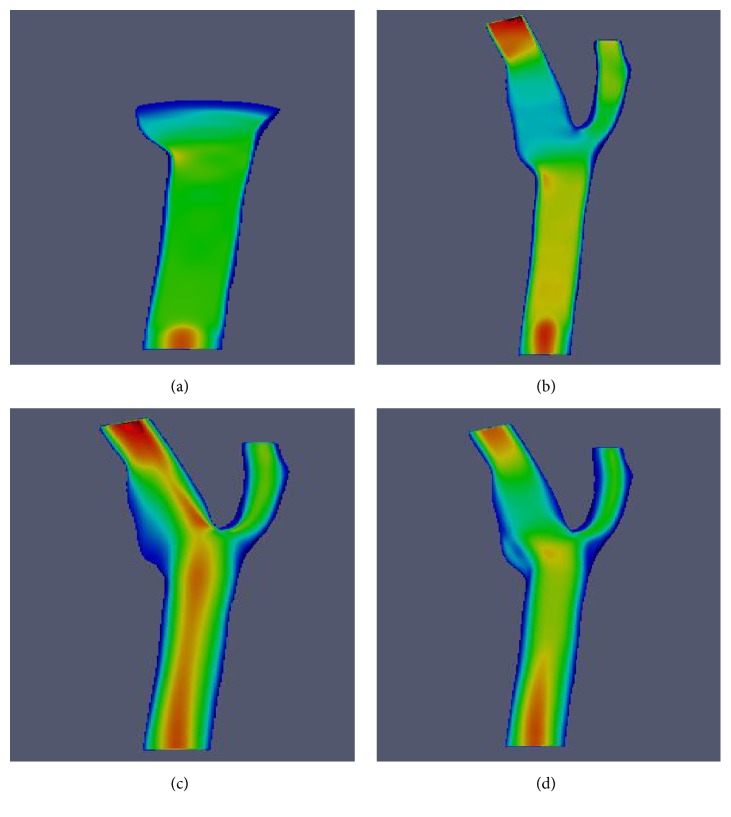
2D Multiphase velocity of blood flow for different time steps.

Figure 21 depicts the 2D multiphase profile of velocity at various time steps. In Figure 21(b), there exists low velocity domain in the aneurysm region as previously described in Figure 20(b). Figure 21(b) shows that higher velocity flow region occurs near the bottom parts of the bifurcation region where high WSS pressure is induced.

### 6.3. Parallel Implementation

**Figure 22:**
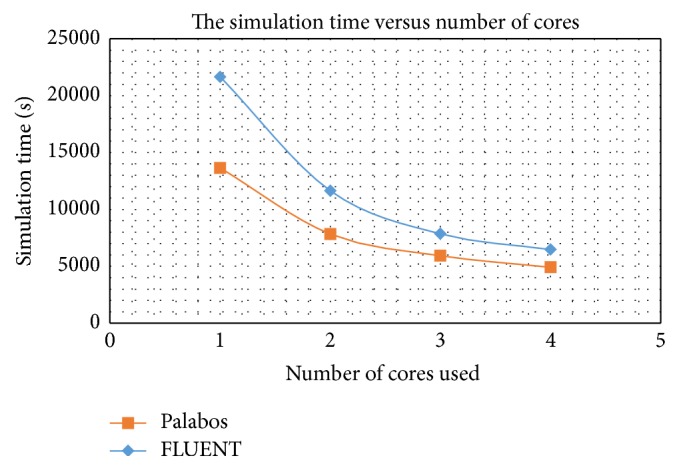
Comparisons of the simulation time versus number of cores for Palabos and FLUENT.

In Table 4, the LBM solver is shown to perform more efficiently than the conventional FVM solver. LBM takes considerably smaller time than FVM to execute the simulation, with 58.79% smaller computation time when applied on single core platform. As seen in Figure 22, the simulation time obtained using FLUENT is relatively similar compared to Palabos as the numbers of cores is increased. Even so, it still imposes around 32.40% and 31.64% differences when 3 and 4 cores are used. The speed-up curve shows smaller gradient as the number of cores is increased from single core up to 4 cores. Similar behavior can be observed for both Palabos and FLUENT. Many reasons could have attributed to this issue, but it is primarily due to the interconnection and communication time between the different cores used. As the number of cores is increased, the tasks will be subdivided into much smaller task resulting to smaller computation time to solve. In the same time, as the numerical of core increases, the interconnection and communication time between the cores will increase substantially. Therefore, it is not worth to add more cores at some point when the computation time became saturated. This is consistent with the findings by Abas and Abdul-Rahman in which it was found that as the number of cores increases for similar applied problem, at some point it will reach saturation point where most of the computation time is spent on the interconnection and communication between individual cores [75].

## 7. Conclusions

In this study, the Lattice Boltzmann method (LBM) for 3D transient blood flow in aneurysm geometry has been validated by comparing the simulation results with those obtained by conventional commercial FVM solver. The LBM solver can provide nearly identical solutions to those of the FV method. Several versions of the LBM were tested and all LBM models showed the same results. The pressure, velocity, and the WSS profile from both methods were compared and demonstrated with good agreement between the different solvers. The maximum percentage difference of pressure for the 15 points of interest come out to a mere 5% difference and the absolute change for the velocity values ranges from 0 to 0.017 ms^−1^. It was also found that the WSS contours for the LBM are observed to be more acceptable compared to a significantly high WSS induced by FVM in the bifurcation region. In addition, this paper evaluates the performance of parallel implementation in each method. From the findings it was shown that LBM is more efficient than the FVM in terms of the parallel computing setup.

The main purpose of this paper is to demonstrate that LBM presents a viable alternative for computation of hemodynamics problems. It is self-evident that the validation of this new method against existing established methodologies has provided an additional platform for CFD analysis in biomedical application. Nevertheless, this study shows that LBM can be considered an alternative solver for computational hemodynamics that can produce results of similar quality compared to those obtained from the Navier-Stokes solvers.

## Figures and Tables

**Table 1:** Weighting functions for D3Q19.

Model	*c*_*s*_^2^	Node no.	Weight
D3Q19	1/3	*f*_0_	1/3
		*f*_1_–*f*_6_	1/18
		*f*_7_–*f*_18_	1/36

**Table 2:** Differences between iliac and cerebral arteries dimensions [29–31].

Artery	Vessel caliber (cm)	Wall thickness (cm)
Iliac artery	1.04	0.076
Cerebral/intracranial artery	0.16–0.77	0.026

**Table 3:** Flow parameters.

Parameters	Value
Density of blood	1060 kg/m^3^
Kinematic viscosity	3.7037 × 10^−6^ m^2^/s

**Table 4:** The tabulated form of simulation time and percentage difference.

Number of cores	PALABOS_time (s)	FLUENT_time (s)	Difference between FLUENT_time (s) and PALABOS_time (s)	Percentage difference (%)
1	13640.2	21660	8019.8	58.79532558
2	7838.8	11640	3801.2	48.49211614
3	5936.6	7860	1923.4	32.39901627
4	4922.4	6480	1557.6	31.64310093
